# Esophageal rupture caused by explosion of an automobile tire tube: a case report

**DOI:** 10.1186/1752-1947-7-211

**Published:** 2013-08-23

**Authors:** Yongkang Yu, Sheng Ding, Yifeng Zheng, Wei Li, Lie Yang, Xiushan Zheng, Xiaoyan Liu, Jianqing Jiang

**Affiliations:** 1 Department of Thoracic Surgery, General Hospital of Chengdu Military Region of PLA, Chengdu 610083, China

**Keywords:** Automobile tire tube, Esophageal rupture, Esophagogastrostomy, Explosion

## Abstract

**Introduction:**

There have been no reports in the literature of esophageal rupture in adults resulting from an explosion of an automobile tire. We report the first case of just such an occurrence after an individual bit into a tire, causing it to explode in his mouth.

**Case presentation:**

A 47-year-old Han Chinese man presented with massive hemorrhage in his left eye after he accidentally bit an automobile tire tube which burst into his mouth. He was diagnosed with esophageal rupture based on a chest computed tomography scan and barium swallow examination. Drainage of empyema (right chest), removal of thoracic esophagus, exposure of cervical esophagus, cardiac ligation and gastrostomy were performed respectively. After that, esophagogastrostomy was performed.

**Conclusions:**

Successful anastomosis was obtained at the neck with no postoperative complications 3 months after the surgery. The patient was discharged with satisfactory outcomes. We present this case report to bring attention to esophageal rupture in adults during the explosion of an automobile tire tube in the mouth.

## Introduction

To the best of our knowledge, esophageal rupture has been rarely reported. A search of the English-language literature using “esophageal rupture” and “automobile tire” revealed no other cases of esophageal rupture in adults caused by biting an automobile tire. Therefore, esophageal rupture may be misdiagnosed because no clinical information and/or guidance are available. In this case report, we present the case of a 47-year-old male motor-repair worker who had an esophageal rupture after he bit an automobile tire tube.

## Case presentation

A 47-year-old male Han Chinese motor-repair worker was admitted to the department of ophthalmology of our hospital due to massive hemorrhage in his left eye after he bit an automobile tire tube at 12:00 a.m. on February 9, 2011. The physical examination revealed extensive rupture of the tarsal plate of his left upper eyelid, and lacrimal canaliculus rupture and bulbar conjunctival hemorrhage were observed. A computed tomography (CT) scan indicated subarachnoid hemorrhage. No abnormality was observed after abdominal ultrasound examination. A chest X-ray indicated pulmonary contusion (60% of the total volume).

**Figure 1:**
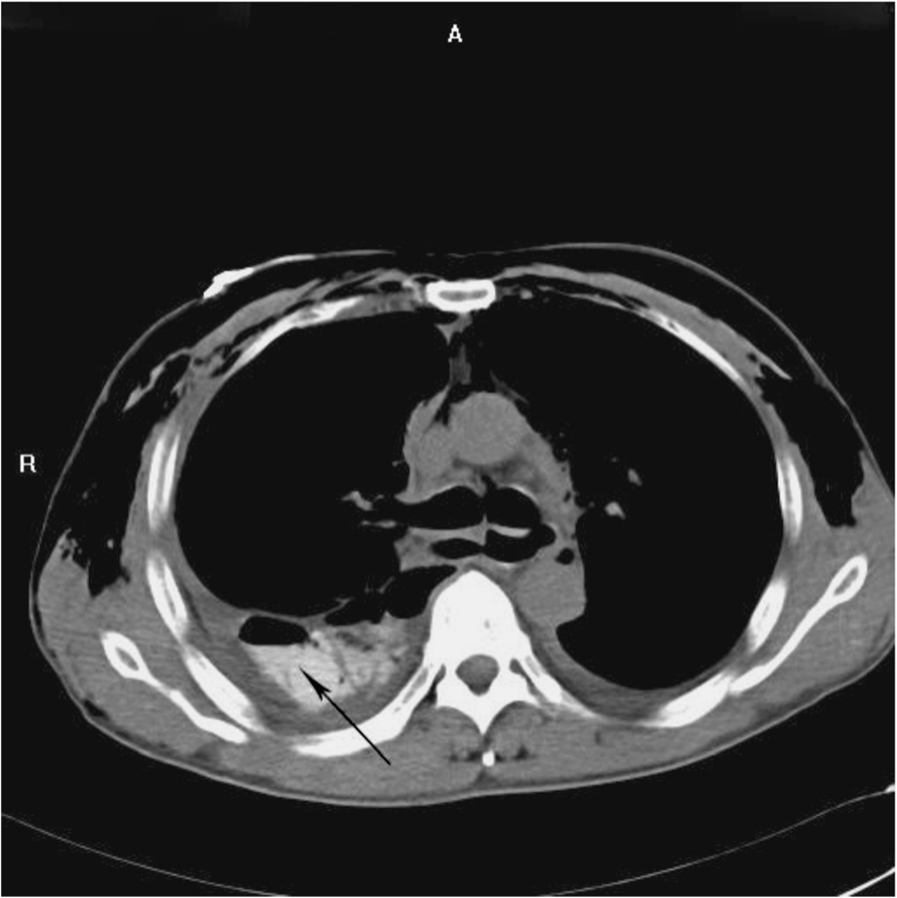
**Chest computed tomography scan and barium swallow examination indicated esophageal rupture.** Arrow: barium entered into thoracic cavity through the ruptured esophagus. A: anterior. R: right.

Closed drainage of his right pleural cavity was performed via the second intercostal space at 4:00 p.m. on February 10, 2011. Sudden chest pain was reported by the patient 12 hours later. A physical examination showed that his heart rate was 120 beats per minute, his temperature was 38°C, his blood pressure was normal, and his respiratory rate was 25 breaths per minute. Chest ultrasound detection was then performed, which revealed severe pleural effusion in his right thoracic cavity. Subsequently, closed drainage of the pleural cavity was performed via the seventh intercostal space, and approximately 800mL of yellow hydrothorax was extracted. A chest CT and barium swallow examination showed an irregular tear in the middle and lower portions of his esophagus (Figure 1). The patient was then transferred to our department of thoracic surgery immediately. For the treatment, drainage of empyema (right chest), removal of thoracic esophagus, exposure of cervical esophagus, cardiac ligation and gastrostomy were performed respectively. Severe inflammation of his right thoracic cavity was observed during the operation. There was a large amount of purulent secretion (approximately 1500mL) in his right thoracic cavity. As exposure of cervical esophagus was noticed during the operation, an ostomy bag (ConvaTec Shanghai Ltd., Shanghai, China) was used to collect the secretions (e.g. saliva) from his esophagus. Meanwhile, gastrostomy feeding and therapeutic antibiotics (10 days) were given. The patient could drink water 72 hours later. The water could directly flow to the ostomy bag. The patient was discharged 10 days later. A successful esophagogastrostomy was obtained 3 months after the first surgery using a gastric tube (4cm in width) constructed from the greater gastric curvature.

## Discussion

A tractor tire has been reported as an etiologic factor of barotrauma especially to the aerodigestive tract [1]. According to previous reports, a sudden and rapid rise in air pressure in the mouth can lead to pressure which would transmit to the laryngopharynx and enter the respiratory passages, resulting in a reflex closure of the glottis [2,3]. As the cricopharyngeus gives way under air pressure, the air pressure gives rise to a sudden distention of the esophagus. The cardia fails to relax because it is involved in the slower, coordinated, reflex act of swallowing, and the dilated esophagus ruptures [4].

Esophageal rupture in children due to the explosion of an automobile tire has been reported [2,5], but no cases have been reported in adults. In our study, the patient was transferred to the department of ophthalmology at first because the doctors speculated that the patient might have an ocular injury. When our patient reported sudden chest pain, a chest CT scan was performed which indicated esophageal rupture. For the treatment of esophageal rupture in children, Kadian *et al*. [2] reported gastrostomy feeds and antibiotics for an 8-year-old child. In another report, Stamm gastrostomy and tube thoracostomy together with broad-spectrum antibiotics were introduced [5]. In this case report, we needed to expose the cervical esophagus during the operation. Because exposure of the patient’s cervical esophagus was noticed during the initial operation, cardiac ligation was performed to prevent the upwards migration of hydrothorax. An ostomy bag was used to collect the secretions (e.g. saliva) in the esophagus, which can prevent thoracic cavity infection. On this basis, the patient could drink water 72 hours after the first operation. In addition, gastrostomy feeding was performed to infuse milk (2000mL per day) for nutritional support. Gastric volume was determined before esophagogastrostomy to check whether gastric atrophy occurred, and a volume of 3000mL was observed, which indicated that the stomach was operating under normal working conditions.

To date, there are still disputes about esophagogastrostomy in the neck or chest after esophageal resection. In a prospective randomized trial aimed to compare sutured neck anastomosis with stapled intrathoracic anastomosis, neck and chest anastomoses after esophageal resection were found to be equally safe [6]. When the stomach was used to replace the esophagus, the anastomosis with the cervical stump tended to be made at the fundus [7]. In our case report, a gastric tube constructed from the greater gastric curvature was applied. Successful anastomosis was obtained in the patient’s neck and he was discharged 10 days later. During the 9 months follow up, normal eating and drinking were attained with no postoperative complications.

## Conclusions

This case report presents the case of a 47-year-old male motor-repair worker with an irregular tear in his esophagus after he bit an automobile tire tube. He was initially speculated to have an ocular disorder due to massive hemorrhage in his left eye. He was diagnosed with esophageal rupture after a chest CT scan and barium swallow examination. Drainage of empyema (right chest), removal of thoracic esophagus, exposure of cervical esophagus, cardiac ligation and gastrostomy were performed respectively. Successful anastomosis was obtained at the neck 3 months later with no postoperative complications. Finally, he was discharged with satisfactory outcomes. We present this case report to bring attention to esophageal rupture in adults during the explosion of an automobile tire tube in the mouth.

## Consent

Written informed consent was obtained from the patient for publication of this case report and accompanying images. A copy of the written consent is available for review by the Editor-in-Chief of this journal.

## Competing interests

The authors declare that they have no competing interests.

## Authors’ contributions

YY wrote the majority of this case report. SD was the main consultant doctor for the management of the patient. YZ and WL performed the procedure of treatment. LY, XZ, and XL contributed to the case history notes used in this case report. JJ was involved in the writing of this manuscript and contributed to the revision of the manuscript. All of the authors read and approved the final manuscript.
